# Relation Between EEG Measures and Upper Limb Motor Recovery in Stroke Patients: A Scoping Review

**DOI:** 10.1007/s10548-022-00915-y

**Published:** 2022-09-22

**Authors:** Giada Milani, Annibale Antonioni, Andrea Baroni, Paola Malerba, Sofia Straudi

**Affiliations:** 1 IIT@Unife Center for Translational Neurophysiology, Istituto Italiano di Tecnologia, Ferrara, Italy; 2 Department of Neuroscience and Rehabilitation, Ferrara University Hospital, Ferrara, Italy; 3 Unit of Clinical Neurology, Department of Neuroscience and Rehabilitation, University of Ferrara, Ferrara, Italy; 4 Battelle Center for Mathematical Medicine and Center for Biobehavioral Health, The Ohio State University, Columbus, OH USA; 5 Department of Neuroscience and Rehabilitation, University of Ferrara, Via Luigi Borsari 46, 44121 Ferrara, Italy

**Keywords:** Upper limb motor outcome, EEG, Stroke, Rehabilitation, Motor recovery

## Abstract

Current clinical practice does not leverage electroencephalography (EEG) measurements in stroke patients, despite its potential to contribute to post-stroke recovery predictions. We review the literature on the effectiveness of various quantitative and qualitative EEG-based measures after stroke as a tool to predict upper limb motor outcome, in relation to stroke timeframe and applied experimental tasks. Moreover, we aim to provide guidance on the use of EEG in the assessment of upper limb motor recovery after stroke, suggesting a high potential for some metrics in the appropriate context. We identified relevant papers (N = 16) from databases ScienceDirect, Web of Science and MEDLINE, and assessed their methodological quality with the Joanna Briggs Institute (JBI) Critical Appraisal. We applied the Preferred Reporting Systems for Systematic Reviews and Meta-Analyses Extension for Scoping Reviews (PRISMA-ScR) Framework. Identified works used EEG to identify properties including event-related activation, spectral power in physiologically relevant bands, symmetry in brain dynamics, functional connectivity, cortico-muscular coherence and rhythmic coordination. EEG was acquired in resting state or in relation to behavioural conditions. Motor outcome was mainly evaluated with the Upper Limb Fugl-Meyer Assessment. Despite great variability in the literature, data suggests that the most promising EEG quantifiers for predicting post-stroke motor outcome are event-related measures. Measures of spectral power in physiologically relevant bands and measures of brain symmetry also show promise. We suggest that EEG measures may improve our understanding of stroke brain dynamics during recovery, and contribute to establishing a functional prognosis and choosing the rehabilitation approach.

**Supplementary Information:**

The online version contains supplementary material available at 10.1007/s10548-022-00915-y.

## Introduction

Cerebral stroke represents one of the leading causes of death and disability worldwide, with important socioeconomic implications and serious consequences on the quality of life of patients and relatives (Guzik and Bushnell 2017). The breadth of neurological and neurocognitive clinical deficit can negatively affect the patient's daily life, with impairment of the upper limb strongly limiting activity levels and social interactions (Kwakkel and Kollen 2007). Recovery after stroke is heterogeneous, depending on size and location of the initial stroke lesion and on the amount and specificity of rehabilitation received (Stinear et al. 2020).

Prediction of recovery after stroke relies on various elements: clinical parameters (i.e. age, type and size of lesion, time since stroke) (Coupar et al. 2012), presence of Motor-Evoked Potentials (MEP) (Escudero et al. 1998) and neuro-radiological parameters (Stinear 2010). An interesting work by Ramsey et al. demonstrated correlations between white matter lesions and alterations in cognitive and behavioural functions, highlighting interactions between specific behavioural deficits and chances of recovery (Ramsey et al. 2017). Assessment of upper limb impairment in stroke patient is important both as a first assessment (Coleman et al. 2017) for potential recovery prediction, and after patients have undergone treatments, as outcome measure. Numerous tests assess upper extremity motor recovery, with the Fugl-Meyer Assessment for Upper Extremity (FMA-UE) considered the gold standard for motor impairment or motor control (Gebruers et al. 2014; Teasell and Hussein 2020). In particular, the FMA arm score at intake is the best predictor of arm recovery and general disability (Gebruers et al. 2014).

Recovery after stroke relies on brain circuitry reorganization and neuroplasticity (morpho-functional reorganization of neural systems) in the first months after stroke damage (Dimyan and Cohen 2011; Hara 2015). Hence, measures of brain dynamics related to motor outcomes are of interest for patient characterization, both as potential biomarkers and as components of multi-modal measures that can inform on the mechanisms of recovery after stroke. Indeed, EEG measures taken after stroke can document the reorganisation of brain areas that support clinical recovery, by revealing changes in inter-hemispheric balance, activity changes in regions linked to the damaged ones, and the reorganisation of body representation maps (Dimyan and Cohen 2011; Hara 2015; Crema et al. 2022). For example, Hummel et al. suggest that motor impairment after stroke might be also linked to an inhibitory action exerted by the healthy motor area on the affected one (Hummel and Cohen 2006). In particular, EEG can be used in measures that aim to combine alterations in electrocortical activity with the patient’s clinical deficits as assessed through clinical rating scales, an approach that has potential clinical significance. Of note, EEG has also been used to investigate the mechanisms of cortical plasticity in pathologies other than stroke, such as infantile cerebral palsy, where research showed that, in not-severely affected patients, the damaged hemisphere reorganises to compensate for the deficit (Inuggi et al. 2018). This of course provided useful suggestions for specific rehabilitation protocols. EEG measures are also used in detecting functional and connectivity changes, allowing study of their implications in the rehabilitation process (Dimyan and Cohen 2011; Hara 2015).

In this context, quantitative analysis of electroencephalography (EEG) signals has been recently introduced in the stroke population, leveraging its high time-scale resolution, which allows to study cortical activity in response to stroke damage, as well as cortical reorganisation (Dimyan and Cohen 2011; Hara 2015). Data shows that after stroke there is an increase in power in the delta (1–4 Hz) and theta (4–8 Hz) frequency bands and a decrease in power in the beta (12–30 Hz) and alpha (8–12 Hz) bands. Parameters such as ratios in the power of these frequency bands are thought to be potential indicators of early ischemic EEG changes (Rabiller et al. 2015).

When combined with data obtained from clinical evaluations, EEG-based quantifiers can contribute to maximizing rehabilitation potential (Bhagat et al. 2020) via patient characterization, supporting prognosis accuracy and facilitating identification of rehabilitation strategies tailored to the functional status of the subject. In this work, we review the literature to synthesize the use of EEG as a clinical instrument for estimation or prognosis of arm motor recovery in stroke patients. This work aims to provide insights into the use of qEEG in the assessment and prediction of upper limb motor recovery after stroke, as some EEG measures seem to have high potential if applied in a tailored way. Due to the significant heterogeneity in this emerging field, a scoping review approach is applied following the Preferred Reporting Systems for Systematic Reviews and Meta-Analyses Extension for Scoping Reviews (PRISMA-ScR) Framework (Tricco et al. 2018).

## Methods

The protocol of this scoping review is pre-registered on Open Science Framework (OSF) with doi 10.17605/OSF.IO/DE98U. We applied the PRISMA-ScR Framework (Tricco et al. 2018). By classifying and describing clinical studies of EEG technologies applied after stroke, we aim to:

- search, identify, and synthesise research into EEG measures as predictors of motor-recovery in upper limb of stroke patients
- provide clinicians with guidance on the use of qEEG in the assessment of upper limb motor recovery after stroke, highlighting the EEG measures that currently seem most promising for this purpose
- identify and map their methodological quality

### Search Strategy

Articles published in peer-reviewed journals, published conference proceedings, and pre-peer review web publications were potentially eligible. Authors GM and AA conducted literature searches of electronic bibliographic databases in Web of Science, PubMed, Science Direct from January 2011 to June 2021 inclusive. The database search was completed on 30 June 2021.

The research strategy incorporates controlled vocabulary and keywords (i.e., EEG, stroke, upper limb), for a compete search strategy, see Supplement A. We allowed for any EEG-based assessment, including, but not limited to, visual analysis of EEG, quantitative EEG (qEEG), continuous EEG monitoring. The study setting could be in hospital, including situations where patients were conveyed to a specialist laboratory from hospital for EEG recording. For the stroke reference standard, any diagnostic process was accepted (i.e., MRI/A, axial computed tomography [CT/A] and/or specialist opinion).

The choice of search strings, although essential for targeting results, could overlook relevant work in the literature. However, using three databases and the optimisation of the search parameters has, in our opinion, compensated for this methodological choice.

### Study Inclusion/Exclusion Criteria

The study population includes patients with ischemic and/or haemorrhagic stroke who performed functional assessment and underwent EEG acquisition that was analysed. Studies that focused mainly or solely on seizures or Transient Ischaemic Attack (TIA) were excluded. We included observational (including case control studies and cohort studies) and interventional studies that applied EEG data as potential predictor to motor recovery. Inclusion Criteria were: (i) reference in English; (ii) reference includes original quantitative data; (iii) study subjects and setting are described in detail; (iv) study includes stroke patients (defined above); (v) study describes the application of EEG technology for motor recovery prediction. Exclusion Criteria were: (i) studies evaluating the predictive power of the EEG in determining the effectiveness of any rehabilitation treatment; (ii) commentaries, editorials and other publication forms without primary data (scoping reviews, systematic reviews).

### Study Selection

**Fig. 1:**
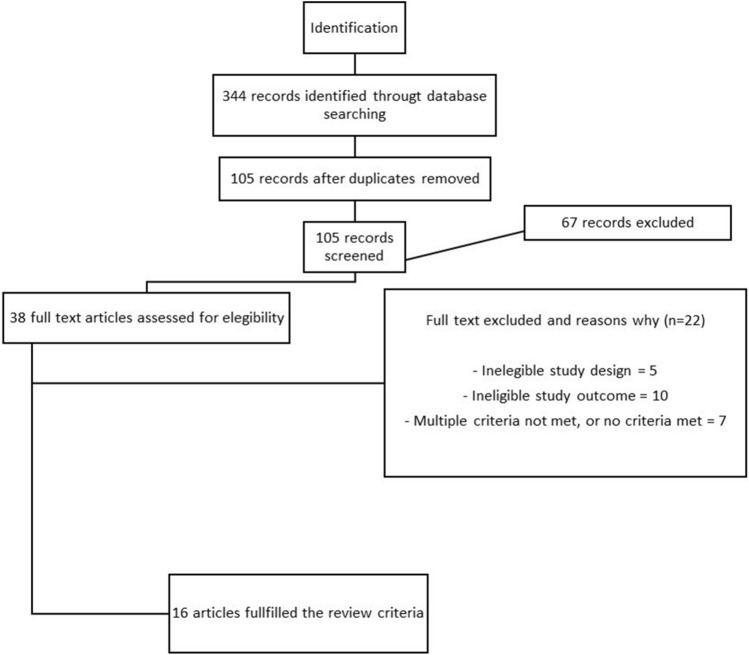
Selection process flow-chart of the included studies

Duplicate articles were excluded. Two members of the study team (GM and AA) reviewed titles and abstracts and selected full text articles to confirm inclusion with arbitration by a third reviewer if required (SS and/or AB). See Fig. 1.

### Data Extraction

Data were independently extracted by two reviewers (GM and AA), with discrepancies resolved via group discussion. A data extraction framework was developed and piloted by the reviewers before use, which included fields for: Key of the Article, Author(s), Year of publication, country of origin, study design, type of stroke, stroke timeframe, study aim, setting, inclusion/exclusion criteria, EEG technology, EEG data processing methodology, rehab measures, population, time from stroke onset to first EEG measure and major findings. To assess the quality of the selected papers, the Joanna Briggs Institute Scale declined according to the type of paper was used (Faculty of Health and Medical Sciences, The University of Adelaide, Australia). As this was a scoping review, there was no a-priori plan for data meta-analysis and a narrative description is provided. Data are presented in tables according to reference standard or outcome measure in order of publication date.

## Results

Databases searches identified 344 articles. After removal of duplicates, 105 abstracts remained. Of these, 67 abstracts did not meet inclusion criteria, i.e., they were studies evaluating the predictive power of the EEG in determining the effectiveness of any rehabilitation treatment, or commentaries, editorials and other publication forms without primary data. The remaining 38 full text articles were assessed (Fig. 1) and 22 articles were excluded: 5 did not meet the study design criterion, 10 did not meet the outcome criterion, and 7 did not meet multiple criteria (ineligible study design and study outcome). After full text review, 16 articles were included for data extraction and quality assessment. Most studies were case–control (n = 9) or cross-sectional (n = 5) studies, although not all specifically used these terms. There were 2 longitudinal studies (Hoshino et al. 2020; Saes et al. 2020). Studies were geographically distributed, with 4 in the Netherlands; 3 in China; 2 each in Japan, Korea, and Austria; and 1 each in USA, Spain, Germany, Israel, and Australia. All the studies were conducted either in acute care, in rehabilitation inpatient setting or in a neurophysiology or kinematics clinic; interestingly, 3 studies used a specially equipped van with dedicated equipment to carry out EEG measurements. The median number of patients was 26.5 (range 9–53). Median time from stroke onset to EEG application was 157.01 days (range 4 to 1074 days), where this information was available (5 studies did not specify this information). Most studies considered chronic (6 months past stroke onset) stroke patient populations (n = 5); to a lesser extent, subacute (1 to 6 months past stroke onset) (n = 5), subacute and chronic (n = 3) and acute (the first 4 weeks after stroke onset) (n = 2) patients; only one study described patients in both acute and subacute phase (Bartur et al. 2019). See Tables 1, 2 and 3 for a summary of the characteristics and results of the included papers.

**Table 1:** Study characteristics of included studies

Unique identifying number	Title	Author	Year of publication	Country of origin	Study design	Quality of study score
1	How does upper extremity Fugl-Meyer motor score relate to resting- state EEG in chronic stroke? A power spectral density analysis	Saes, M, et al	2019	Holland (Amsterdam)	Case–Control study	10/10
2	Changes in mu and beta amplitude of the EEG during upper limb movement correlate with motor impairment and structural damage in subacute stroke	Bartur, G, et al	2019	Israel (Tel Aviv)	Case–Control study	8/10
3	Event-Related Desynchronization During Mirror Visual Feedback: A Comparison of Older Adults and People After Stroke	Fong, KNK, et al	2021	China (Kong Kong)	Case–Control study	6/10
4	An approach for assessing stroke motor function ability using the similarity between electroencephalographic power spectral densities on both motor cortices	Ha, J, et al	2018	Korea	Cross-sectional study	4/8
5	EEG Biomarkers Related With the Functional State of Stroke Patients	Sebastian-Romagosa, M, et al	2020	Spain (Barcelona) and Austria (Schiedlberg)	Case–Control study	10/10
6	Relationship between upper limb function and functional neural connectivity among motor-related areas during recovery stage after stroke	Hoshino, T, et al	2020	Japan	Longitudinal study	6/8
7	Resting State Functional Connectivity Is Associated With Motor Pathway Integrity and Upper-Limb Behavior in Chronic Stroke	Hordacre, B, et al	2020	Australia (Adelaide)	Case–Control study	9/10
8	Parietofrontal network upregulation after motor stroke	Bönstrup, M, et al	2018	Germany and USA	Case–Control study	10/10
9	Are early measured resting-state EEG parameters predictive for upper limb motor impairment six months poststroke?	Saes, M, et al	2021	Holland (Amsterdam)	Cross-sectional study	6/8
10	Is Resting-State EEG Longitudinally Associated With Recovery of Clinical Neurological Impairments Early Poststroke? A Prospective Cohort Study	Saes, M, et al	2020	Amsterdam, Netherlands	Longitudinal study	8/11
11	Electroencephalographic Phase Synchrony Index as a Biomarker of Poststroke Motor Impairment and Recovery	Kawano, T, et al	2020	Osaka, Japan	Case–Control study	10/10
12	EEG-based motor network biomarkers for identifying target patients with stroke for upper limb rehabilitation and its construct validity	Chen, C, et al	2017	Taiwan, China	Case–Control study	9/10
13	Poor motor function is associated with reduced sensory processing after stroke	Campfens, S. Floor, et al	2015	Enschede, Netherlands	Cross-sectional study	6/8
14	EEG patterns of subacute stroke patients performing motor tasks correlate with motor functional outcome: Preliminary results	Park, W, et al	2016	Korea	Cross-sectional study	5/8
15	Abnormal functional corticomuscular coupling after stroke	Chen, X, et al	2018	Hebei, China	Case–Control study	8/10
16	Relationship Between Electrical Brain Responses to Motor Imagery and Motor Impairment in Stroke	Kaiser, V, et al	2012	Graz, Austria	Cross-sectional study	5/8

**Table 2:** Clinical and demographic characteristics of the study population and EEG acquisition

Unique identifying number	Sample size	Type of stroke	Stroke time frame	Time from stroke onset to first EEG measure	EEG acquisition	EEG montage
1	21 patients (15 males; mean age, 60.6; range, 48–77)	Not specified	Chronic (54.7 months)	6 months	Resting state	64 channels
2	14 patients (8 males, mean age, 58 ± 15.8); 13 healthy subjects	Ischemic or hemorrhagic	Acute, Subacute (45,1 days)	13–103 days	Auditory cue motor task	64 channels
3	11 patients (5 males; mean age, 56.1 ± 14.35); 13 healthy subjects (7 males; mean age, 55.54 ± 5.68)	Not specified	Chronic (6 months after stroke onset)	35.8 ± 22.93 months	Auditory cue motor task ± Mirror	64 channels
4	14 patients (11 males, mean age, 53.4 ± 7.0)	Not specified	Chronic	Not specified	Visual cue motor task	64 channel
5	36 patients; 32 healthy subjects	Not specified	Subacute, Chronic	4 days	Visual-auditory cue motor task	16 channels
6	24 patients (mean age, 62 ± 12)	Not specified	Subacute	4 and 8° weeks	Resting state and Motor task	5 channels
7	36 patients (26 males, mean age, 64.4 ± 11.1; range, 43–93); 25 healthy subjects (17 male, mean age, 67.3 ± 6.7; range, 52–77)	Ischemic or hemorrhagic	Chronic (3.6 ± 2.7 (Stroke MEP +) 5.0 ± 3.0 (Stroke MEP-)	6 months	Resting state	64 channels
8	30 patients (19 males, mean age, 65 ± 13); 19 healthy subjects (10 males, mean age, 64.8 ± 11.1)	Not specified	Subacute (104 ± 17 days)	104 ± 17 days	Visual cue motor task	63 channels
9	39 patients	Ischemic	Acute (12.3 ± 5.8 days)	12.3 ± 5.8 days	Resting state	62-channel
10	41 patients (mean age, 67 ± 11)	Ischemic	Subacute (within 3 weeks)	13 ± 5 days	Resting state	62-channel
11	40 patients (mean age, 69.8 ± 13.8); 22 healthy subjects (mean age, 66.9 ± 6.5)	Ischemic	Acute (interval of > 2 weeks after stroke onset)	36.9 ± 11.8 days	Resting state + Open/Close eyes	19-channel
12	53 patients (15 males; mean age, 59.21 ± 12.40)	Ischemic or hemorrhagic	Subacute, Chronic (5.10 ± 4.63 months)	Not specified	Auditory cue motor task	32-channel
13	11 patients (10 males; mean age, 60,45 ± 12.80)	Ischemic	Subacute (within 6 months)	Not specified	Motor task	64-channel
14	9 patients (mean age, 58.3 ± 5.9)	Ischemic	Subacute (1 month after stroke onset)	22.9 ± 7.1 days	Visual-auditory cue motor task	64-channel
15	16 patients (9 males; mean age, 50.50 ± 15.41; range, 28–72); 8 healthy subjects (4 males; mean age, 60.5 ± 6.26; range, 52–70)	Ischemic or hemorrhagic	Subacute, Chronic (2–23 months)	Not specified	Motor task	64-channel
16	29 patients (15 males; mean age, 58 ± 15)	Ischemic or hemorrhagic	Chronic (4 ± 4 months)	Not specified	Motor task (motor imagery and execution)	61-channel

**Table 3:** Study results including rehabilitative outcome measures and biomarkers EEG

Unique identifying number	Outcome measures / rehab measure	EEG measures	Results
1	FMA	DAR, BSI	Higher BSI in chronic stroke patients compared to controls, most pronounced in delta and theta frequency bands. In delta and theta band, BSI was significantly negatively associated with FM-UE. DAR showed no differences between groups nor association with FM-UE. BSIdir showed increased power in the affected versus the unaffected hemisphere
2	BBT, FMA	ERD	The ERD magnitude in the high-mu and low-beta bands, measured over the affected hemisphere, correlated significantly with residual motor function in the paretic upper limb as measured by standard clinical tests
3	FTHUE, ARAT, FMA, WMFT	AI, ERD, ERSP	Comparing the effect of task versus group in contralateral and ipsilateral motor areas showed a significant effect of task condition at the contralateral motor area in the high beta band (17–35 Hz) at C3. High beta ERD was larger in the contralateral vs ipsilateral hemisphere in both study groups. The magnitude of low beta (12–16 Hz) ERD in patients with stroke was more suppressed in contralesional C3 under the no mirror compared to that of the covered mirror condition and similarly more suppressed in ipsilesional C4 ERD under the no mirror compared to that of the mirror condition. Correlation analysis revealed that the magnitude of ERSP correlated significantly with arm severity in the low and high beta bands in patients with stroke, and a higher AI in the low beta band was associated with higher arm functioning under the no-mirror condition. There was a shift in sensorimotor ERD toward the contralateral hemisphere as induced by mirror visual feedback accompanying unimanual movement in both stroke patients and healthy controls
4	FMA	PSD, PLV	EEG power spectral densities were similar between the ipsilesional and contralesional area. This feature was significantly correlated with FMA score of the affected hand. Brain activity of patients with low motor function appeared in ipsilesional and contralesional areas, whereas brain activity of patients with high motor function was confined to the ipsilesional area
5	FMA, BI, FTRS, MAS, BBT, 9HPT, TPDT, SRQ, MOCA	BSI, LC	Significant differences in BSI between healthy subcortical stroke group, and between the healthy and cortical-and-subcortical stroke group. No significant differences found between the healthy group and the cortical stroke group. BSI in the healthy group showed statistical differences based on gender. In the stroke group, the correlation between BSI and FMA-upper extremity was also significant. The correlation between the BSI and the FMA-lower extremity was not significant. Similarly, LC calculated in the alpha band showed significative correlation with FMA of upper extremity and FMA of lower extremity. Other important significant correlations between LC and functional scales were observed
6	FMA	FCs	FMA scores evaluated at 4 W (33 ± 24 (SD)) improved by 8 W (42 ± 23). FCs in alpha and beta bands calculated between electrodes in the ipsilesional hemisphere correlated negatively with FMA score at 4 W after stroke. FCs obtained at 4 W could be used to predict the FMA score 8 W after stroke
7	FMA, ARAT, HGST	RSFC	Βeta frequency interhemispheric sensorimotor RSFC greater for MEP+ stroke participants compared with MEP−. Significant positive correlation between beta RSFC and upper limb behavior that appeared to be primarily driven by the MEP+ group. A hierarchical regression identified that the addition of beta RSFC to measures of CST integrity explained greater variance in upper limb behaviour
8	9HPT, FMA	SMR	Parietofrontal coupling significantly stronger in patients compared to controls. Motor network coupling generally increased during the task in both groups. Task-related coherence between parietal and primary motor cortex in the affected hemisphere showed increased connectivity across a broad range of sensorimotor rhythms. Parietofrontal task-induced coupling was significantly and positively related to residual impairment in the 9HPT performance and grip force. Parietofrontal motor system integration during visually guided movements is stronger in the stroke-lesioned brain
9	FMA, NIHSS, ARAT, EmNSA, MI-UE, EHI, Bamford Classification	DAR, BSI	BSI calculated over the theta band was the strongest EEG-based predictor regarding FM-UE-w26. BSI-theta remained a significant predictor when added to a regression model including FM-UE-baseline, increasing explained variance from 61.5 to 68.1%. Higher BSI-theta values- predicted more upper limb motor impairment 6 months after stroke
10	NIHSS, FMA, ARAT, EmNSA, MI-UE	DAR, BSI, BSIdir	Spectral characteristics showed a gradual normalization over time, within and beyond 12 weeks poststroke. Significant within- and between-subject associations with NIHSS were found for DAR of the affected hemisphere and BSIdir-delta. BSIdir-delta also demonstrated significant within- and between-subject associations with FM-UE. Changes in spectral characteristics are not restricted to the time window of recovery of clinical neurological impairments
11	FMA	PSI	The interhemispheric PSI (alpha band) between the primary motor areas (M1s) was lower in patients than in controls and was selectively correlated with FM-UE. In contrast, the PSI (theta band) on the contralesional M1 was higher in patients than controls and was selectively correlated with FM-UE gain. The latter correlation was significant in severely impaired patients (FM-UE ≤ 10)
12	FMA, TEMPA, WMFT	Frequency- and connection-specific parameters and frequency-specific spectrum dynamics in 5 core motor cortices	Predictive statistical model. Features of beta and gamma or theta network combined provided the best classification accuracy. The predictive value and the sensitivity of these biomarkers were 81.3% and 90.9%, respectively. Subcortical lesion, the time poststroke and initial WMFT score were identified as the most significant clinical variables affecting classification accuracy of this predictive model. Moreover, 12 of 14 controls were classified as having favourable recovery
13	FMA, EmNSA, MAS	PCC	All subjects showed significant contralateral PCC in affected and non-affected wrist tasks. Subjects with poor motor function had a reduced contralateral PCC compared to subjects with good motor function in the affected wrist tasks. Amplitude of significant PCC did not differ between subjects with good and poor motor function
14	FMA	ERD, bilateral beta-band spectral power values	Beta band spectral power in bilateral motor cortex after physical upper limb movement correlated significantly with FMA scores
15	STM	CMC	Compared to healthy controls, stroke patients had abnormally reduced coherence in the EEG-BB (EEG to biceps brachii) combination and increased coherence in the EEG-DT (EEG to deltoid) combination. Compared to synkinetic stroke patients, separate ones exhibited higher gamma band coupling during stage 1 of the motor task and higher beta band coupling during stage 2 of the motor task in EEG-BB combination, but lower at beta-band during stage 2 in EEG-DT combination
16	ESS, MRC, MAS	ERD, ERS, LC	Higher impairment was related to stronger ERD in the unaffected hemisphere and higher spasticity was related to stronger ERD in the affected hemisphere. Both were related to a relatively stronger ERS in the affected hemisphere. For the LC of ERS during motor execution (ME) and motor imagery (MI) of the affected hand, a significant relationship with the degree of impairment (ME) and spasticity (MI) exhibited by patients was identified. Higher spasticity and impairment were associated with a relatively stronger ipsilesional ERS

*FMA* Fugl-Meyer Assessment, *FM-UE* Fugl-Meyer Upper Extremity, *ARAT* Action Research Arm Test, *MI-EU* Motricity Index-Upper extremity, *MRC* Medical Research Council Scale for Muscle Strength, *WMFT* Wolf Motor Function Test, *BBT* Box and Block Test, *9HPT* Nine-Hole Peg Test, *SRQ* Shoulder Rating Questionnaire, *HGST* Hand Grip Strength Test, *TEMPA* Upper extremity performance test for the elderly, *STM* Shang Tian Min test system, *FTH-UE* Functional Test for the Hemiplegic Upper Extremity, *mAS* Modified Ashworth Scale, *EmNSA* Erasmus MC modification of the Nottingham Sensory, *NIHSS* National Institutes of Health Stroke Scale, *FTRS* Fahn Tremor Rating Scale, *BI* Barthel Index, *TPDT* Two Point Discrimination Test, *MoCA* Montreal Cognitive Assessment, *EHI* Edinburgh Handedness Inventory, *BC* Bamford Classification, *ESS* European Stroke Scale, *ERS/D* Event-Related Synchronization/desynchronization, *ERSP* Event-Related Spectral Potentials, *PSD* Power-Spectral Density, bilateral beta-band power, *DAR* delta/alpha ratio, *BSI* Brain Symmetry Index, *LC* Laterality Coefficient, *PCC* Position Cortical Coherence, *FC* Functional Connectivity, *CMC* Measures of Cortico-Muscular Coherence, *PLV* Phase Locking Value, *PSI* Phase Synchrony Index

### EEG Procedures

Visual input was administered during EEG acquisition (n = 2), or EEG recording occurred during resting state (n = 4), or motor tasks (n = 3) or the administration of acoustic stimuli (n = 2) or visual-auditory cue motor tasks (n = 2). We found a single study for each of the following categories: resting state and motor tasks, resting state in open/closed eyes conditions and auditory task with mirror task. See Supplement B.

### Clinical Outcomes

Several outcome measures were considered for arm motor recovery assessment. The prevailing one was the FMA (n = 14), less common was the Action Research Arm Test (ARAT) (n = 4). Some studies used other indices: Motricity Index-Upper extremity (MI-EU), Medical Research Council Scale for Muscle Strength (MRC) and Wolf Motor Function Test (WMFT) (n = 2 each); Box and Block Test (BBT), Nine-Hole Peg Test (9HPT), Shoulder Rating Questionnaire (SRQ), Hand Grip Strength Test (HGST), Upper extremity performance test for the elderly (TEMPA), Shang Tian Min test system (STM) and Functional Test for the Hemiplegic Upper Extremity (FTH-UE) found in one study each. See Supplemental D.

### EEG Measures

**Fig. 2:**
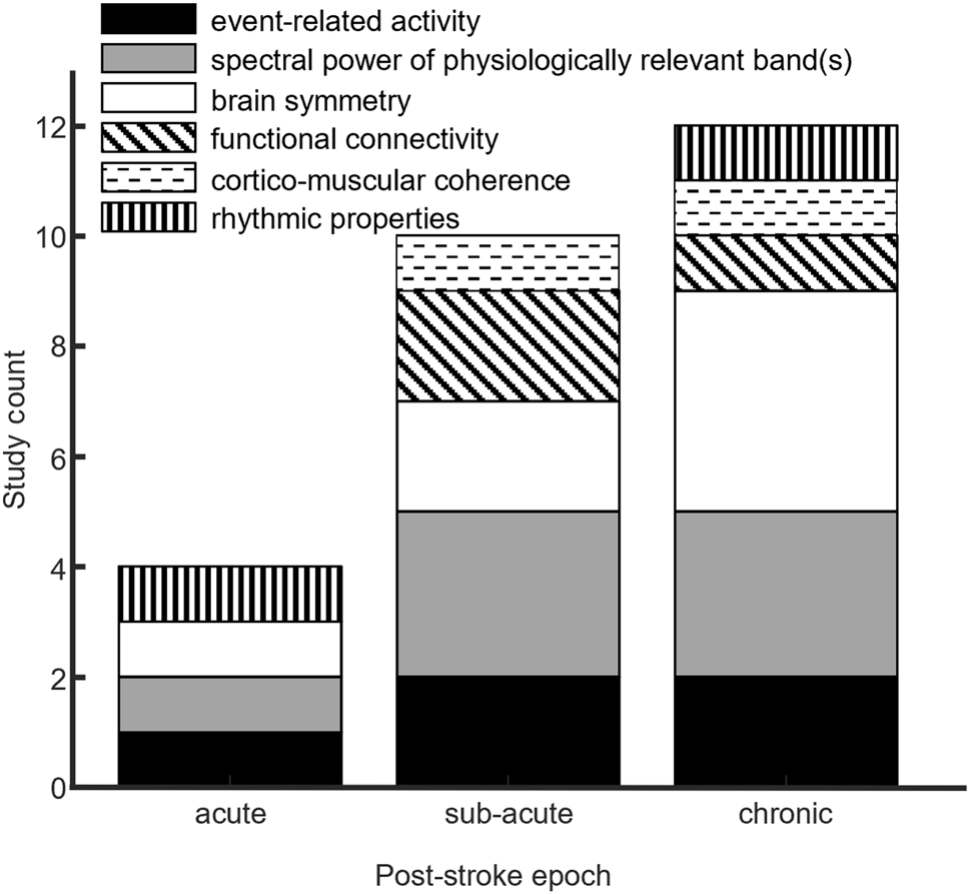
Included studies according to stroke timeframe and EEG measures

EEG analysis in the context of motor recovery stroke research has focused on multiple types of quantifiers (see Fig. 2). Quantifiers were applied in relation to an action (event related, often linked to motor tasks or visual tasks) or as stationary descriptors. Rhythmic activity was studied as power densities or as phasic relations (e.g., Phase Locking Value, Phase Synchrony Index). See Supplement C. We identify the following categories of EEG measures:

- Event-related measures: synchronization/desynchronization (ERS/D) (n = 4) and Event-Related Spectral Potentials (ERSP) (n = 1)
- Measures of spectral power in physiologically relevant bands: spectrogram (n = 1), power-spectral density (PSD) (n = 1), bilateral beta-band power (n = 1) and mu and beta band R-means (n = 1); delta/alpha ratio (DAR) (n = 3)
- Measures of bran symmetry: Brain Symmetry Index (BSI) (n = 5), Laterality Coefficient (LC) (n = 2)
- Measures of functional connectivity: Coherence Analysis (CA) (n = 1), Functional Connectivity (FC) (n = 2).
- Cortico-Muscular Coherence (CMC) (n = 1);
- Measures of rhythmic properties (such as phase coordination): Phase Locking Value (PLV) (n = 1) and Phase Synchrony Index (PSI) (n = 1).

In the following, we report the relation between these measures and motor outcome organized by the time (since stroke) in which the EEG was acquired: acute, subacute, or chronic. When a specific measure is applied to a rhythmic band, we will hyphen the band after the measure (e.g., ERD in the beta band is abbreviated with ERD-beta).

### Event-Related Measures

Two types of event-related potential measure were identified in the literature: ERS/D and ERSP. ERS/D is the short-lasting potentiation/attenuation of rhythms, often studied within the alpha or beta band, either unilaterally or bilaterally; while ERSPs measure cortical potentials linked to a movement.

ERD was mainly found in beta band in relation to visual and auditory stimulation, either during the stimulus or preceding it (Park et al. 2016; Bartur et al. 2019; Fong et al. 2021). In one study, during movement of the affected hand, chronic stroke patients with less impairment assessed by higher MRC values showed higher contralesional ERS-mu, whereas patients with higher impairment showed higher ipsilesional ERS-mu (Kaiser et al. 2012). In another study, beta band spectral power in the bilateral motor cortex after upper limb movement negatively correlated with FMA scores in subacute patients (Park et al. 2016). Consistently, another study found that ERD-high-mu and ERD-low-beta of the affected hemisphere correlated significantly (positively) with FMA in the paretic upper limb of acute/subacute patients (Bartur et al. 2019). Similarly, using a mirror/no mirror condition in a Mirror Visual Feedback (MVF) task with chronic patients, Fong et al. found that ERSP power showed a significant negative correlation with arm severity in the low and high beta bands, and that MVF-induced attenuation of ERD-low-beta was greater on the contralateral hemisphere, compared to the ipsilateral one (Fong et al. 2021).

### Measures of Spectral Power in Physiologically Relevant Bands

Studies leveraged three types of spectral power quantifiers: PSD, which measures the power distribution in the frequency domain; DAR, estimating the relation between high and low frequencies during resting state; and rhythm-based estimates. These include the Sensory-Motor Rhythm (SMR, a 13–15 Hz wave found in EEG leads placed near sensory-motor cortices) (Bönstrup et al. 2018) and combinations of power in multiple bands used to build predictive statistical models, for example combining beta and gamma estimates (Chen et al. 2017).

A decrease of DAR in the affected hemisphere (DARAH) reflective of improvement of global neurological impairments was found in the subacute phase (Saes et al. 2020) but not in the acute (Saes et al. 2021) and chronic stroke phases (Saes et al. 2019).

In a subacute phase study, task-related coherence between parietal and primary motor cortex in the lesioned hemisphere showed increased connectivity across a broad range of SMR, and parieto-frontal task-induced coupling was significantly and positively related to residual impairment (Bönstrup et al. 2018).

A study estimating predictive models that combined measures of power in multiple frequencies showed that, in the subacute and chronic phase, combining beta estimates with either theta or gamma estimates provided the best classification accuracy (92%). In particular, subcortical lesion, time post stroke and initial WMFT score were identified as the most significant clinical variables in this predictive model (Chen et al. 2017).

A chronic stroke study showed similar EEG PSD-beta between the ipsi- and contra-lesional hemispheres, which was significantly correlated with the FMA of the affected hand; this was driven by bilateral beta in patients with low motor function and ipsilesional-only beta in patients with high motor function during movement of the affected hand (Ha et al. 2018). Of note, another study conducted in the chronic phase (not detected by the research strategy used), showed that a higher delta power bilaterally correlated with better motor status. The authors suggest that this might reflect adaptive plasticity related to attentional processing, task complexity, or cognitive control (Cassidy et al. 2020).

### Measures of Brain Symmetry

After a stroke, interhemispheric balance is altered, with greater activity of the injured hemisphere compared to the healthy one. This can be functionally interpreted as an attempt to recover the functions compromised by brain damage (Lefaucheur et al. 2014) or as a form of maladaptation that correlates with a poor clinical outcome, since excessive activity of the damaged motor cortex might hinder the vicarious and reorganising activity carried out by the healthy hemisphere (Thibaut et al. 2017). Measures of EEG symmetry among hemispheres can be meaningful quantifiers of this imbalance. These include: (1) BSI, which compares the power spectra between the two hemispheres during resting state; (2) directional BSI (BSIdir), which specifies which of the two hemispheres shows larger power; (3) Laterality Coefficient (LC), which is calculated using ERD/ERS; and (4) Asymmetry Index (AI) which computes the difference between band activity in the two hemispheres (similar to BSI, but measured during ERD).

In the acute stroke phase, higher BSI-theta predicted lower FMA 26 weeks after stroke (Saes et al. 2021). In subacute stroke, a decrease of BSIdir-delta reflected improvement of global neurological impairments and was also specifically associated with upper-limb motor recovery early post stroke (Saes et al. 2020). In the chronic phase, BSI-delta and BSI-theta were significantly negatively associated with FMA and BSIdir in the delta, theta and alpha band showed increased power in the affected versus the unaffected hemisphere (Saes et al. 2019). In later stroke phases, significant relations were documented between BSI-alpha and FMA and between LC-alpha and several clinical scales (Sebastián-Romagosa et al. 2020). Moreover, a significant relation of LC of ERS to the degree of impairment during motor execution and spasticity during motor imagery of the affected hand was identified (Kaiser et al. 2012). A higher AI-low-beta was also associated with higher arm functioning under a specific task condition in the chronic phase (Fong et al. 2021).

### Measures of Functional Connectivity

The literature also covers coherence analysis, including Positional Cortical Coherence (PCC), which represents the agreement between mechanically evoked perturbations and EEG as a measure of afferent pathway integrity, and FC, which identifies statistical (undirected) associations among spatially distinct brain areas.

In the subacute phase, subjects with poor motor function had reduced contralateral PCC compared to subjects with good motor function in the affected wrist tasks (Campfens et al. 2015). Moreover, the FC-alpha and FC-beta, calculated over the ipsilesional hemisphere, correlated negatively with FMA at four weeks after stroke (Hoshino et al. 2020). Hordacre et al. (2020) showed that FC-beta among hemispheres during sensorimotor resting state (RSFC) was larger in chronic stroke patients that showed MEPs (MEP+) compared to those who did not show MEP (MEP−). FC-beta was also positively correlated with upper limb function. Of note, a recent paper (which was not part of our database since its publication was after our search end date) has highlighted a prominent role of coherence, rather than power, in predicting early motor recovery after acute stroke. Specifically, only the low beta band recorded on M1 demonstrated negative associations with motor recovery, highlighting the maladaptive nature of beta coherence between ipsilesional M1 and ipsilesional parietal and controlateral temporal and supplementary motor area in early stroke recovery (Cassidy et al. 2021). Interestingly, another work of the same group (not found by the research strategy used) showed that delta band coherence with ipsilesional M1 was related to greater injury and poorer motor status in a subacute timeframe (Cassidy et al. 2020). Thus, data suggests that coherence in different bands changes across stroke timeframes, suggesting a potential relevance of coherence in describing the time-based evolution of motor recovery after stroke.

### Measures of Cortico-Muscular Coherence

CMC measures the coordination between cortex and muscle activity, interpreted as assessing the cortical control of muscle activation. One study found that stroke patients had abnormally reduced cortico-muscolar coherence, which was positively related to motor function as measured by STM (Chen et al. 2018).

### Measures of Rhythmic Properties

Studies employed measures of rhythmic properties, including PLV, which quantifies phase coordination between two time-series, and the interhemispheric PSI, which quantifies zero-phase locking between the two hemispheres.

One study found that in the acute stroke phase, the interhemispheric PSI-alpha between the primary motor areas (M1) was lower in stroke patients compared to controls, and positively correlated with FMA. In the same study, the PSI-theta on the contralesional M1 was higher in patients than controls, and correlated with FMA gain (Kawano et al. 2020).

A study in chronic stroke patients quantified the correlation between PSD-beta in the ipsilesional and contralesional M1 (as a measure of similarity), and found that it correlated significantly with the FMA of the affected hand (Ha et al. 2018).

### Quality Assessment

All the studies included in the review were evaluated using the Joanna Briggs Institute (JBI) Critical Appraisal (Faculty of Health and Medical Sciences, the University of Adelaide, Australia). To account for different study types (cross sectional, case control or cohort) we organize quality assessment in a checklist that evaluates enrolment process, exposure, management of confounding factors, outcome assessment and statistical analysis. Selection of participants was described with sufficient detail in the majority of the included studies. Two studies recruited a convenience sample: Fong et al. (2021) recruited subjects from community self-help groups without knowledge on lesion site, Hordacre et al. (2020) recruited chronic stroke survivors from the community resulting in an uneven sample size between MEP+ and MEP− groups, with possible implications on subgroup analysis. Furthermore, the demographics of the older adult control participants recruited by Fong et al. (2021) did not match with those in the stroke group. One study did not provide a detailed description of subjects included and lacked specification on the level of motor impairment (Ha et al. 2018); one study did not specify their inclusion criteria (Park et al. 2016). A recruitment bias could be identified in Chen et al. (2018) who included subacute and chronic stroke without considering spontaneous recovery within 6 months post-lesion. All included studies gave detailed rationale and description of intervention, except for Kaiser et al. (2012) where task execution was not uniform across patients due to reduced compliance to recording session duration.

Across the reviewed literature, the leading cause of quality score reduction is the lack of identification and management of confounding factors. Only seven studies (Saes et al. 2020; Bönstrup et al. 2018; Chen et al. 2017; Saes et al. 2019; Sebastián-Romagosa et al. 2020; Hordacre et al. 2020; Kawano et al. 2020) considered potential confounders and measured them. Outcome measurement was completed in a valid and reliable way for all the included studies, considering inter-subject and inter-trial variability typical of EEG recordings. All the studies reported detailed statistical analysis in their methods section; accuracy of analysis was not considered in our evaluation, delegating such assessment to the peer review process, which all considered studies underwent. Study quality scores are reported in Table 1.

## Discussion

We aim to provide clinicians with guidance on the use of qEEG in the assessment of upper limb motor recovery after stroke. This information could contribute to a rehabilitation medicine tailored to the individual patient. EEG in stroke rehabilitation can be used either as a concurrent measure of motor recovery (Kaiser et al. 2012; Bartur et al. 2019; Fong et al. 2021; Bönstrup et al. 2018; Saes et al. 2019; Ha et al. 2018; Sebastián-Romagosa et al. 2020; Hordacre et al. 2020; Park et al. 2016; Campfens et al. 2015; Kawano et al. 2020) or as a predictive measure, with studies correlating acute EEG with motor recovery measures 3–6 months later (Chen et al. 2017; Park et al. 2016; Saes et al. 2020, 2021; Hoshino et al. 2020; Chen et al. 2018).

We found 16 studies, and a large variance in study paradigms and EEG analysis strategies, which undermines our ability to reach data-driven conclusions on qEEG likelihood to predict motor recovery. In the literature, event-related activation, spectral power in physiologically relevant bands and indices of brain symmetry were the most investigated measures.

Among event-related measures, ERS/D in alpha and beta band were reported in the different post-stroke phases, making it possible to evaluate, even with inter-study variability, their evolution overtime. ERS is reduced in the affected hemisphere, regardless of stroke recovery phase (Fong et al. 2021; Kaiser et al. 2012; Ezquerro et al. 2019; Stępień et al. 2011) and ERD correlates with motor outcome, (Bartur et al. 2019; Ezquerro et al. 2019) in both subacute and chronic stroke. Of note, we did not find any studies examining ERD in the acute phase, probably due to challenges in achieving communication and cooperation early after stroke. Therefore, we suggest that ERD measures are appropriate in the mid-to-late stages of stroke recovery. Current ERD data seem to indicate the activation of compensatory physiological mechanisms in both hemispheres. Further studies could evaluate ERDs at long delays after stroke to assess the possible recovery of lost functions by the affected hemisphere or their definitive shift to the healthy one.

Spectral power measures in physiologically relevant bands have also provided useful insights into the reorganisation of brain activity following injury. Among the PSD measures, parieto-frontal coupling, which may indicate sensorimotor integration, is related to residual impairment (Bönstrup et al. 2018). Also, beta-band PSD is similar in the two hemispheres in patients with low motor function, but biased toward the ipsilesional area in those with high motor function (Ha et al. 2018). Other studies have shown that high-frequency rhythms are also associated to motor impairment, and an excess of beta power in the affected central cortical region is associated with poor motor function (Thibaut et al. 2017). These data suggest that in patients with good motor outcome lesioned areas reorganize rapidly, whereas when outcome is worse support is needed from contralateral areas. This could imply that recovery can be promoted in the injured areas through various methods (e.g., personalized non-invasive brain stimulation or arm rehabilitation) that inhibit the activity of the healthy areas, in order to recruit damaged areas back into the plastic recovery process, particularly in patients with mild motor deficit (Murase et al. 2004). On the other hand, in patients with severe motor deficit, when the affected areas are too compromised, it may be useful to evaluate the stimulation of the healthy hemisphere, in order to promote its intervention in the vicariation of the lost functions in the contralateral hemisphere (Di Pino et al. 2014). In comparison, DAR did not provide information on prognosis in the acute and chronic phase (Saes et al. 2019, 2021), while in the subacute one a decrease in DARAH correlates with better long-term prognosis (Saes et al. 2020). These data suggest that the key phase for predicting recovery (at least in relation to this EEG measure) is the subacute one: once the acute damage has passed, the neural reorganisation mechanisms are activated and determine the long-term outcome; in the chronic phase, outcomes are already stabilised and the possibility to predict prognosis is reduced. It is noteworthy, however, that according to Cassidy et al., a higher delta power bilaterally correlated with better motor status, with authors suggesting a possible dependency of this factor on adaptive plasticity related to attentional processing, task complexity, or cognitive control (Cassidy et al. 2020). Thus, the presence of a predominant delta rhythm appears to have different prognostic significance depending on the stage of the stroke investigated. Further studies may clarify the neurophysiological relevance of this pattern in the delta band in the post-stroke timeframe.

Interestingly, one study showed that subcortical injuries are more significant than cortical ones in predicting recovery, underlining the role of distant connections between various brain regions and basal ganglia, fundamental in motor regulation (Chen et al. 2017).

Finally, measures of brain symmetry during resting state showed high potential as biomarkers. BSI, particularly in the theta and delta band, correlates with post stroke motor recovery (Saes et al. 2020, 2021, 2019), and similarly LC-alpha band correlates with FMA and other recovery scales (Sebastián-Romagosa et al. 2020). The correlation between BSI and motor outcome is particularly relevant in the case of subcortical lesions, probably because of the hypothesized importance of short-and-long distance network changes in recovery (Sebastián-Romagosa et al. 2020). This suggests that hemispheric imbalances can reflect severity of injury and predict recovery.

Other categories of EEG measures that we found in the literature were under-explored, limiting our ability to draw general conclusions. FC measures can describe reorganization of connections developing after injury, although the need for high density EEG montages to calculate FC measures limits their applicability in current clinical settings (Hoshino et al. 2020; Hordacre et al. 2020). Similarly, CMC measurements are extremely innovative in their multi-modal assessment of brain and body dynamics, but require high skills and patient compliance, which could be hard to obtain, especially in acute phases (Chen et al. 2018; Guo et al. 2020).

Considering the limitations of the current literature, it is hard to predict exactly which EEG measures are going to be most appropriate to future studies. However, we predict that some EEG measures have high potential for impact, when applied to the appropriate question. In the following, we outline our perspective on which EEG measures are most likely to prove useful in light of the current literature, organized by stroke stage. In the acute phase, most studies have focused on resting-state measures, consistent with the known challenges in collecting motor task data and patient compliance. Given the importance of the interhemispheric imbalance to evaluate the reorganisation of the damaged areas or the intervention of the contralateral hemisphere to vicariate the lost functions (Saes et al. 2021), we suggest the evaluation of the BSI in this timeframe. BSI may provide useful insights on the most appropriate non-invasive brain stimulation protocol in the early phase, i.e. inhibition (Murase et al. 2004) or excitation (Di Pino et al. 2014) of the healthy hemisphere, considering both the clinical condition and the interhemispheric imbalance. Notably, BSI’s applicability also extends to later stages, which is why we ideally suggest studies of longer duration and larger sample sizes to assess its evolution in relation to the motor outcome. The subacute and chronic phase allow acquisition of motor tasks, and data so far indicates a good correlation between these measures and upper limb motor recovery, in particular assessed by FMA. The DARH (Saes et al. 2020) appears very promising in the subacute phase, mainly to stratify patients after the earliest stage of damage and make predictions about recovery in order to tailor neurorehabilitation treatment. Structural and functional neuroimaging could also be very useful, as the subcortical connections between non-adjacent areas seem to be of particular importance in stroke motor recovery (Chen et al. 2017).

Finally, in the chronic phase, ERD (Kaiser et al. 2012), BSI (Saes et al. 2019) and LC (Sebastián-Romagosa et al. 2020) appear most promising. They are useful to investigate how brain activity reorganises itself after a significant amount of time from the acute injury. Although at this stage the damage is stabilised and chances of recovery are reduced, knowledge of brain activity changes can provide useful suggestions on prevention of maladaptive modifications and choice of non-invasive brain stimulation approaches aimed to reverse them. At this stage, since more information is needed on how these measures with great potential for clinical relevance evolve in relation to stroke time and motor outcome, we suggest that future studies intensify the simultaneous use of several EEG measures in chronic stroke, leveraging the likely increased patient cooperation.

Finally, the study of these neurophysiological measures could provide useful information on the brain mechanisms that mediate the effects of various therapies used in the rehabilitation field. Therefore, by exploring the neurophysiological mechanisms that are most activated by specific rehabilitation treatments, it will be possible to select the most appropriate behavioral rehabilitation treatment for the patient, based on the alterations revealed by the EEG, such as mirror therapy, functional electrical stimulation and robotics (Jia et al. 2022; Jaafar et al. 2021; Daly and Ruff 2007).

### Limitations

This review was limited by the small number of papers on the topic that met inclusion criteria, the variability in stroke timeframe, study design and EEG measures investigated, as well as the small number of patients enrolled in the studies.

Our choice to systematically organize our review in relation to stroke phase led to some studies cross-contributing, as they considered multiple periods from stroke onset. We also emphasize a lack of standardized methodology on EEG montage, analysis and acquisition setting. This scoping review also highlights the wide variability on clinical tests and scales used to assess changes in clinical conditions over time, which compounds the limitation of interpretability in scoring using clinical tests. A further limitation is the use of strings for the research in datasets, which can lead to lack of completeness, with relevant studies not captured (e.g., Thibaut et al. 2017; Cassidy et al. 2020).

## Conclusions

This scoping review aims to provide the state of the art on EEG-derived measures that are useful in stroke arm rehabilitation. Several metrics have been found to be related to arm motor recovery across all stages of motor rehabilitation (acute, subacute and chronic), despite the small number and heterogeneity of available studies. Efficacious metrics include: brain symmetry (i.e. BSI, especially in the acute phase), spectral power (i.e. DARH, particularly in the subacute timeframe), and event-related measures (i.e. action observation ERS/ERD, especially, but not only, in the chronic phase); which were the most explored EEG measures with a potential role in studying changes in brain dynamics, interhemispheric imbalance and reorganization processes that occur after stroke. These data may provide useful suggestions aimed at stratifying patients according to their chances of recovery in order, to tailor neurorehabilitation treatments and develop non-invasive brain stimulation protocols that (1) promote the recovery of damaged areas, (2) modulate the intervention of healthy ones and (3) counteract maladaptive changes. We propose that this scoping review can be considered as a starting point for scientists and clinicians for selecting appropriate EEG measures to assess spontaneous and rehabilitation-induced motor recovery.

## Supplementary Information

Below is the link to the electronic supplementary material.

Supplementary file1 (DOCX 27 kb)

## Data Availability

Data sharing not applicable to this article as no datasets were generated during the current study.
