# Supplementary material for: Relation Between EEG Measures and Upper Limb Motor Recovery in Stroke Patients: A Scoping Review
Source: Brain Topogr. 2022 Sep 22;35(5-6):651–66. doi: 10.1007/s10548-022-00915-y (PMC9684227; doi:10.1007/s10548-022-00915-y)
Supplement: Supplementary file 1 — Supplementary file1 (DOCX 27 kb) [file 10548_2022_915_MOESM1_ESM.docx]

**SUPPLEMENTARY MATERIAL**

**Supplement A. Search strategy (strings used to search databases)**

1. TI=(stroke OR "cerebrovascular accident" OR CVA OR "brain infarction") OR AB=(stroke OR "cerebrovascular accident" OR CVA OR "brain infarction") AND TI=(eeg OR electroencephalogra* OR "brain waves" OR "brain rhythm") OR AB=(eeg OR electroencephalogra* OR "brain waves" OR "brain rhythm”) AND TI=("upper limb" OR "upper arm" OR "upper extremity" OR arm) OR AB=("upper limb" OR "upper arm" OR "upper extremity" OR arm)
2. (stroke OR "cerebrovascular accident" OR "brain infarction") AND (eeg OR electroencephalogram OR "brain waves") AND ("upper limb" OR upper arm OR "upper extremity")
3. ((((((((stroke[MeSH Terms]) OR (stroke[Title/Abstract])) OR ("cerebrovascular accident"[Title/Abstract])) OR ("cerebrovascular infarction"[Title/Abstract])) OR (CVA[Title/Abstract])) OR ("brain infarction"[Title/Abstract])) OR ("cerebrovascular disorder"[Title/Abstract])) AND (((((Electroencephalography[MeSH Terms]) OR (Electroencephalography[Title/Abstract])) OR (EEG[Title/Abstract])) OR (Electroencephalogra*[Title/Abstract])) OR ("brain waves"[Title/Abstract]))) AND (((((Upper Extremity[MeSH Terms]) OR (Upper Extremity[Title/Abstract])) OR (Upper Limb[Title/Abstract])) OR ("upper arm"[Title/Abstract])) OR ("arm"[Title/Abstract]))

**Supplement B. Detailed experimental setting**

| **Unique identifying number** | **Type of task** | **Task description** | **Number of repetitions/ Duration of task** |
| --- | --- | --- | --- |
| 1 | Resting state | EEG recording at rest | 5 consecutive trials of one minute each |
| 2 | Auditory cue motor task | 3-second wrist extension activity and a 3- second rest period. | 16 cycles |
| 3 | Auditory cue motor task +/- Mirror | Three task conditions: in front of a mirror (viewing a reflected image of the unaffected arm, with the affected arm at rest), with no mirror and in front of a covered mirror. The loudspeaker presented one stimulus (the “ting” tone) every 5 s. Each task condition consisted of two identical blocks consisted of 40 trials with consecutive wrist flexion and extension movement. | 40 trials |
| 4 | Visual-cue motor task | Patients had to perform a grasping movement with affected hand according to the monitor cues; after the participants performed the movement, the device remained in the same position for 1 second, and then moved back to the original position within 2 seconds. Participants were trained before the experiment. At the beginning of each run, task instructions were shown on the screen. Then, a fixation cross was displayed, and participants were instructed to stare at the cross without moving their head. In the few seconds that followed, participants waited for a task cue while gazing at the fixation cross. When the fixation cross changed to a circle paired with a beep sound, the participants performed a motor task for 2000 ms. The training device stopped for 1000 ms, and the circle changed back to the fixation cross. The training device then returned to its starting position for 2000 ms. During this return period, participants were instructed not to exert control on the device. | 42 times |
| 5 | Visual-Auditory Cue motor task | Patients, after an auditory preparation cue, saw an arrow pointing to the left or right and heard “left” or “right” via headphones; these simultaneous cues direct the patient to imagine dorsiflexion of the left or right wrist until the relax command is presented auditorily. | 25 sessions |
| 6 | Resting state + Motor task | Patients performed resting and finger task EEGs; during the task EEG session, they tried to move their fingers on the affected side randomly. Sessions with synkinesia or mirror movement observed visually of the other hand were excluded. | 60 seconds (at rest, with closed eyes and keeping relaxed but awake); not speficied during the finger task |
| 7 | Resting state | Patients performed resting state keeping their eyes open and looking straight ahead at a fixation point (refraining from speaking or moving or actively engaging in any cognitive or mental tasks). | 3 minutes |
| 8 | Visual cue motor task | Patients (avoiding eye movements) performed an isometric visually guided whole hand grips with the paretic hand; there were two conditions: keeping the force constant across the group and keeping the task effort constant across the group. The begin of each grip as well as continuous feedback about the applied force were provided visually by the appearance and vertical level of a horizontal bar on a screen. | Each condition was recorded with 20 repetitions of 9 seconds |
| 9 | Resting state | EEG recording at rest | Five 1-minute trials, with sufficient rest in between |
| 10 | Resting state | EEG recording at rest | The duration of the full EEG protocol was dependent on patient’s ability to perform tasks. Including preparation of the patient this took between 45 minutes, in case only resting-state EEG was measured, and 2 hours, in case all tasks were performed |
| 11 | Resting-state + Open/Close eyes | Participants had to relax and alternately open and close their eyes for 30 seconds | 10 times, 5 minutes |
| 12 | Auditory cue motor task | For every 10 successful trials, participants took a short break of 1–10 minutes. All participants were instructed not to blink in the first 2 seconds of each movement. | 80 trials |
| 13 | Motor task | Patients performed a motor task with the affected arm and with the non-affected arm in which they exerted a constant wrist flexion torque; when they were unable to maintain a stable wrist flexion torque with the affected wrist or unable to return to the target torque once the exerted torque decreased, they performed the relax task, in which they held the handle without exerting a torque. | Eight trials of 40 seconds |
| 14 | Visual-Auditory cue motor task | Patients performed three runs of 16 sessions, composed of task notice, motor task block and rest period; a motor task block consisted of five trials with each trial (60 trials for each) consisting of relaxation, motor task, stay and return periods and four motor tasks (passive, active, motor imagery and stand-by) were used. The participants performed the experiment three times: at enrolment, two weeks after the first experiment and eight weeks after the second one. | 16 sessions |
| 15 | Motor task | Patients performed the arm lift task to measure "flexion synergy"; at the 2nd second after data recording, they were to slowly lift their hands to the chest within 3 s according to the tip (phase 1) and then perform a movement to the chest maintained for 3 s (phase 2) | 20 sessions with 60 pauses |
| 16 | Motor task (motor imagery and execution) | The session was divided into runs. Each trial was temporally determined by a cursor appearing in the low center of the screen and moving toward the top at a constant velocity on a straight trajectory. During rest trials, the patient was asked to watch the cursors trajectory. During motor task trials, a green rectangle appeared at the top of the screen. The patient was asked to start performing the cued motor task when the cursor reached the green rectangle and to continue until the end of the trajectory. The command sequence was randomized. Patients were asked to either execute or imagine simple movements (grasping and complete finger extension) with the affected and unaffected hand in separate runs. Each run was dedicated to a different motor task; the run sequence was randomized across patients. | 30 trials for each run (15 rest and 15 motor trials per run) |

**Supplement C. EEG Measures**

| **EEG Measures** | | **Description** | **Measure** |
| --- | --- | --- | --- |
| **DAR** | Delta/alpha ratio | Relationships between high and low frequencies | Spectral power |
| **DTABR** | Delta theta/alpha beta ratio | Relationships between high and low frequencies | Spectral power |
| **BSI** | Brain symmetry index | Compares power spectra between the two cranial hemispheres and provides the magnitude of their asymmetry | Brain symmetry |
| **ERD** | Event Related Desynchronization | Is the short-lasting attenuation or blocking of rhythms within the alpha (beta) band. ERD is found during but also before visual stimulation. | Event-related |
| **LC** | Laterality coefficient | Is a parameter calculated using the ERD/ERS changes in the mu wave | Brain symmetry |
| **ERSP** | Event-Related Spectral Potentials | Measures average dynamic changes in amplitude of the broad band EEG frequency spectrum as a function of time relative to an experimental event. | Event-related |
| **RSFC** | Resting state functional connectivity | Measures temporal correlation of spontaneous signal among spatially distributed brain regions. | Functional connectivity |
| **PSI** | Interhemispheric phase synchrony index | The left and right hemisphere oscillations exhibited phase-locking with a phase-lag near zero degrees. The index of synchronization was strongest when these oscillations had large amplitude. | Rhythmic properties |
| **Spectrogram** |  | Signal strength based on time and frequency. | Spectral power |
| **PSD** | EEG power spectral densities | Represents the power distribution of EEG series in the frequency domain. It measures the power according to the frequency only, you have information on which frequencies are most present. | Spectral power |
| **CMC** | Cortico-muscolar coherence | Study the mechanism of cerebral cortex's control of muscle activity. | Cortico-Muscolar Coherence |
| **PLV** | Phase locking Value | Is a measure of the propensity for two time series signals to maintain constant phase separation with each other over a period of time. | Rhythmic properties |
| **PCC** | Position-cortical coherence | Is the agreement between mechanically evoked perturbations and electroencephalography, as a measure of afferent pathway integrity. | Functional connectivity |
| **FCs** | Functional connectivity | Identify statistical (undirected) associations among spatially distinct brain areas. | Functional connectivity |
| **SMR** | Sensorimotor rhythm | An oscillation showing of synchronized electric brain activity during idle movement. | Spectral power |
| **AI** | Asymmetry Index | Difference between left and right band activity | Brain symmetry |

**Supplement D. Rehab Measure**

| **Rehab Measure** | | **Description** |
| --- | --- | --- |
| **FMA** | Fugl-Meyer Assessment | Assess motor functioning, balance, sensation and joint functioning |
| **MRC** | Medical Research Council (MRC) Scale for Muscle Strength | Assess muscle strength from Grade 5 (normal) to Grade 0 (no visible contraction). |
| **BBT** | Box and Block Test | Measure unilateral gross manual dexterity. |
| **mAS** | Modified Ashworth Scale | Measure the increase of muscle tone |
| **FTRS** | Fahn Tremor Rating Scale | Is a tremor rating scale |
| **BI** | Barthel Index | Measure a person's performance in activities of daily living. |
| **9HPT** | Nine-Hole Peg Test | Measure finger dexterity |
| **SRQ** | Shoulder Rating Questionnaire | Assess shoulder disorders |
| **MoCA** | Montreal Cognitive Assessment | Is a cognitive screening test |
| **ARAT** | Action Research Arm Test | Assess coordination, dexterity and functioning |
| **HGST** | Hand Grip Strength Test | Assess the strength of the muscles that participate in the "grip" of the hand |
| **9NHP** | Nine Hole Peg test | Measure finger dexterity |
| **NIHSS** | National Institutes of Health Stroke Scale | Quantify the impairment caused by a stroke. |
| **EmNSA** | Erasmus MC modification of the Nottingham Sensory | Somatosensory assessment measure |
| **MI-UE** | Motricity Index  - Upper extremity | Measure limb strength |
| **EHI** | Edinburgh Handedness Inventory | Assessment and analysis of handedness |
| **Bamford Classification** |  | Divides people with stroke into four different categories, according to the symptoms and signs with which they present. |
| **TEMPA** | Upper extremity performance test for the elderly | Performance-based test to assess upper extremity function |
| **WMFT** | Wolf Motor Function Test | Quantifies upper extremity (UE) motor ability through timed and functional tasks |
| **STM** | Shang Tian Min (STM) test system | Assess the movement of ‘uplift the affected hand to reach the ipsilateral ear’ to distinguish the synkinetic and separate phases for stroke |
